# CSPG Is a Secreted Factor that Stimulates Neural Stem Cell Survival Possibly by Enhanced EGFR Signaling

**DOI:** 10.1371/journal.pone.0015341

**Published:** 2010-12-14

**Authors:** Muly Tham, Srinivas Ramasamy, Hui Theng Gan, Ashray Ramachandran, Anuradha Poonepalli, Yuan Hong Yu, Sohail Ahmed

**Affiliations:** Neural Stem Cell Laboratory, Institute of Medical Biology, Singapore, Singapore; University of Southern California, United States of America

## Abstract

Understanding how autocrine/paracrine factors regulate neural stem cell (NSC) survival and growth is fundamental to the utilization of these cells for therapeutic applications and as cellular models for the brain. *In vitro*, NSCs can be propagated along with neural progenitors (NPs) as neurospheres (nsphs). The nsph conditioned medium (nsph-CM) contains cell-secreted factors that can regulate NSC behavior. However, the identity and exact function of these factors within the nsph-CM has remained elusive. We analyzed the nsph-CM by mass spectrometry and identified DSD-1-proteoglycan, a chondroitin sulfate proteoglycan (CSPG), apolipoprotein E (ApoE) and cystatin C as components of the nsph-CM. Using clonal assays we show that CSPG and ApoE are responsible for the ability of the nsph-CM to stimulate nsph formation whereas cystatin C is not involved. Clonal nsphs generated in the presence of CSPG show more than four-fold increase in NSCs. Thus CSPG specifically enhances the survival of NSCs. CSPG also stimulates the survival of embryonic stem cell (ESC)-derived NSCs, and thus may be involved in the developmental transition of ESCs to NSCs. In addition to its role in NSC survival, CSPG maintains the three dimensional structure of nsphs. Lastly, CSPG's effects on NSC survival may be mediated by enhanced signaling via EGFR, JAK/STAT3 and PI3K/Akt pathways.

## Introduction

*In vivo*, neural stem cells (NSCs) reside in tightly regulated microenvironments known as the stem cell niche. This niche consists of supporting niche cells, extrinsic signals, membrane-bound molecules and the extracellular matrix (ECM). The niche protects stem cells from differentiation signals, apoptotic stimuli and excessive proliferation which can result in cancer [1]. *In vitro*, NSCs can be propagated along with lineage restricted neural progenitors (NPs) as neurospheres (nsphs) or adherent cultures. The nsph conditioned medium (nsph-CM) contains cell-secreted factors that have been shown to increase NSC/NP survival and proliferation [2]–[3]. Nsph-CM also regulates cell fate decisions [4]–[5]. Beside soluble factors, proteoglycans within the ECM are known to act as reservoirs for growth factors [6].

Proteoglycans are proteins with covalently-bound sulfated glycosaminoglycans (GAGs). In the central nervous system (CNS), proteoglycans carry mainly chondroitin sulfate (CS) or heparan sulfate (HS) GAGs [6]. During development, CSPGs regulate cell division [7]–[8], neural crest cell migration [9]–[10] and axon path finding [11]–[14]. In addition, there is increasing evidence that CSPGs regulate NSC/NP proliferation. NSCs/NPs secrete CSPGs including phosphacan, aggrecan and neurocan [15]. NSCs/NPs expressing the DSD-1-proteoglycan (also known as phosphacan) showed increased nsph formation and neurogenesis [16], whereas disruption of CS-GAGs with chondroitinase ABC (chABC) treatment reduces NSC/NP proliferation, secondary nsph formation and neurogenesis. ChABC injected into the embryonic ventricular zone disrupts the cellular architecture of this region and inhibits NSC/NP proliferation [17]. However, the exact role of cell-secreted CSPG remains unclear. Does it affect NSCs, NPs or both and what signaling pathways does CSPG utilize for its effects on NSCs/NPs?

In the present work, we used nsph cultures containing both NSCs and NPs to evaluate the role of nsph-CM on NSC survival. We show by mass spectrometry that nsph-CM contains CSPG, ApoE and cystatin C. The nsph stimulatory effect of nsph-CM can be attributed to CSPG and ApoE but not cystatin C. We show that exogenous CSPG added to NSCs/NPs at clonal densities increases nsph number and size. Using clonal analysis and assays for self-renewal and multipotency, we show that CSPG stimulates specifically NSC survival. In addition, CSPG also enhances the survival of embryonic stem cell (ESC)-derived NSCs. ChABC treatment disrupts the 3-dimentional (3D) structure of nsphs, suggesting a role for CSPG in maintaining nsph integrity. Lastly, we show that CSPG is likely to increase NSC survival via enhancement of the epidermal growth factor receptor (EGFR), Janus kinase/signal transducers and activator of transcription 3 (JAK/STAT3) and phosphoinositide-3-kinase/Akt (PI3K/Akt) signaling pathways.

## Materials and Methods

### Isolation and culturing of NSCs/NPs

All animal experiments were approved by the Biomedical Research Council Singapore, Institutional Animal Care and Use Committee (IACUC #080392) in accordance with national guidelines. NSCs/NPs were isolated from embryonic (E14.5) C57BL/6 mice. Dissociated cortical cells were seeded at 2×10^5^ cells/ml in NSC growth medium (GM) [Dulbecco's Modified Eagle's Medium (DMEM)/F-12, N2 supplement, 20 ng/ml EGF and 1% penicillin/streptomycin (all from Invitrogen)]. Cells were grown at 37°C and 5% CO_2_ atmosphere in a humidified incubator. Nsphs were passaged every 5–7 days. Bulk density cultures were at 2×10^4^ cells/ml and low density cultures were at 2×10^3^ cells/ml (equivalent to 350cells/cm^2^). We estimate that under low density culture conditions approximately 98% of nsphs are clonal based on the following calculation. Based on a recent publication, the estimated aggregation rates for cells cultured at 1×10^3^ cells/ml (250cells/cm^2^) and 5×10^3^ cells/ml (1250cells/cm^2^) are 1.6% and 8% respectively, when cells were not disturbed during the culturing period [18]. Thus there is a five-fold increase in aggregation rate with a five-fold increase in cell density. For our experiments, the cell density is 1.4-fold higher than the lower density used by Coles-Takabe et al. (2008). In addition, our cells were also not disturbed during the culturing period, thus the aggregation rate in our cultures is estimated to be 2.2% (i.e. 1.4-fold higher than the 1.6% aggregation rate for cells cultured at 1×10^3^ cells/ml). This indicates that approximately 98% of the nsphs were likely to be clonal. For hydrogel cultures dissociated cells were plated at 2.5×10^3^ cells/ml in a 1.2% hydrogel/GM solution. For adherent cultures, dissociated cells were plated at 1×10^4^ cells/ml on 0.1% poly-L-lysine (PLL; Sigma) coated plates. Growth curves were generated using the CellTitre Glo cell viability assay (Promega).

### Nsph-CM

Nsph-CM was collected on day five. Nsph-CM and GM were fractionated into proteins greater than (Fraction A) and less than (Fraction B) 30 kDa using Amicon filters (Millipore). The fractions were digested with trypsin (Promega) in 50 mM ammonium bicarbonate (pH 8.0) and 2% acetonitrile overnight at 37°C. The digested peptides were analyzed using a Q-STAR liquid chromatography mass spectrometer and compared with the same fractions from GM. The six sub-fractions were obtained by elution through a liquid chromatography column with a water stationary phase and an acetonitrile mobile phase, and separated into individual factions based on elution time. The fractions were decontaminated and reconstituted back into GM to the original concentration for activity analysis. Digestion of nsph-CM was carried out with 5 mU/ml chABC (Sigma) overnight at 37°C followed by heat inactivation at 100°C for 5 min. ApoE (Calbiochem) was used at 50 nM and RAP (Innovative Research) at 5 µM.

### CSPG and inhibitors

Since purified forms of CSPG e.g. DSD-1-proteoglycan, is not commercially available, we used proteoglycan from bovine nasal septum (Sigma). This preparation consist of 86% chondroitin sulfate, 8% protein, 6% keratan sulfate and less than 1% hyaluronic acid [19]. Unless stated otherwise, all experiments with CSPG (Proteoglycan from bovine nasal septum; Sigma) and CS-GAGs were done in low-density cultures (2×10^3^ cells/ml), while all CSPG inhibitors were tested in bulk-density cultures (2×10^4^ cells/ml). ChABC, sodium chlorate, 4-methylumbelliferyl-β-D-xyloside and keratanase (all from Sigma), PD168393, LY294002, AG490, PD98059 and Y27632 (Calbiochem) and C3 (Cytoskeleton) were used at the stated concentrations. CSPG, CS-A & -C (Sigma), -B, -D & -E (US Biological) and KS (US Biological) were used at 50 µg/ml. All reagents were added at time of cell plating and left for the duration of the culture. Nsph formation was assessed after 5 days by manually counting and sizing all the nsphs in the well.

### ESC derived NSCs

D3 mouse ESCs were grown on mouse embryonic feeder (MEF) layer in ESC medium [DMEM containing 15% fetal bovine serum (FBS), β-mercaptoethanol, non-essential amino acids, glutamine (all Invitrogen) and 1,000 U/ml leukemia inhibitory factor (Chemicon)]. After the removal of MEF, ESCs were seeded at 10^5^ cells/ml in NSC growth medium. Spheres were passaged every 7 days and their numbers assessed after 21 days.

### Differentiation and immunocytochemistry

Single nsphs from low density cultures were transferred to each well of a 50-well cover glass (Sigma) coated with PLL (0.01%) and laminin (10 µg/ml; Invitrogen). Nsphs were differentiated for five days in differentiation medium [DMEM/F-12, N2, 1% penicillin/streptomycin and 0.5% FBS (Invitrogen)]. Cells were stained with mouse IgM anti-O4 (1∶300; Chemicon), mouse IgG_2a_ anti-Tuj1 (βIII-tubulin; 1∶250; Covance) and rabbit IgG anti-GFAP (1∶1000; Dako). The secondary antibodies were Alexa Fluor antibodies 488 anti-mouse IgM, 594 anti-mouse IgG_2a_ and 647 anti-rabbit IgG (1∶500; Invitrogen). Cells were counter stained with DAPI (Invitrogen) and scored based on staining and morphology.

### Western Blot

Dissociated cells were plated in medium without EGF for 3 hours then stimulated with EGF (20 ng/ml), CSPG (50 µg/ml) or CSPG and EGF for the length of time stated. Alternatively, nsphs generated with/without CSPG were harvested after 6 days in culture. Proteins were extracted and transferred to PVDF membrane as previously described [20]. Membranes were immunoblotted with antibodies to the following proteins: phosphotyrosine (PY20) and EGFR (BD Pharmingen); phospho-EGFR (Tyr 1068), phospho-STAT3 (Tyr 705), STAT3, phospho-Akt (Thr308), Akt (Cell Signaling).

### Data analysis

Results are presented as mean ± SEM. Data were analyzed with paired Student T-test. Relative gene expression was calculated using the ABI Relative Quantitation Algorithm (Applied Biosystems). Multiple sets of data were combined by a stratified t-test [21]. Growth curves and inhibitor dose-dependent curves were fitted by GraphPad Prism software. Images were taken with the Olympus point scanning FV-1000 confocal microscope.

## Results

### Nsph-CM stimulates nsph formation

**Figure 1:**
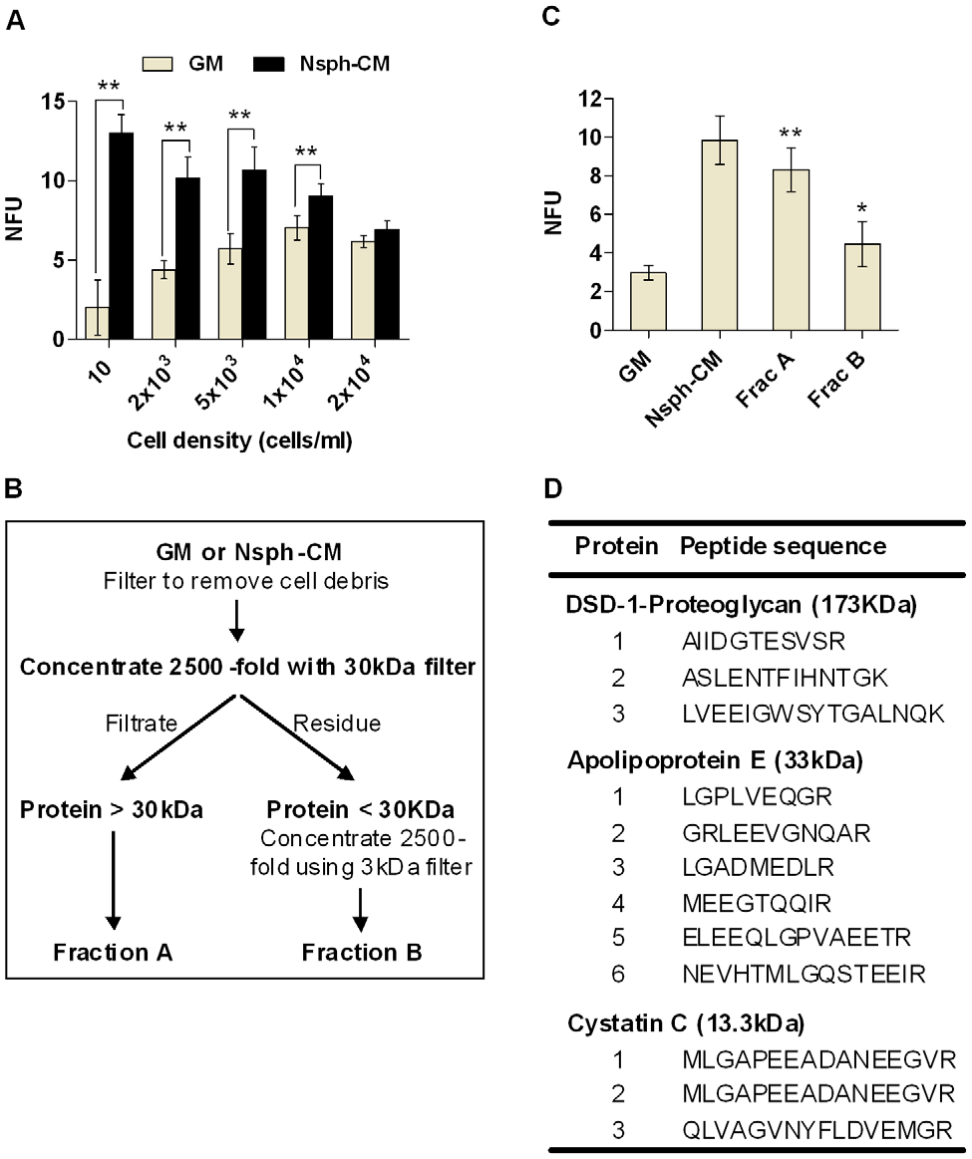
Identification of CSPG, ApoE and Cystatin C in nsph-CM. (**A**) Cells were plated at different densities (between 10 cells/ml to 2×10^4^ cells/ml) in growth medium (GM) or neurosphere-conditioned medium (nsph-CM) and nsph forming units (NFUs) measured after five days of culture. NFU is the number of nsph formed divided by number of cells seeded x 100. The cell density of 10 cells/ml represents 1 cell per well. The data are presented as the mean ± SEM with *n*≥6 (from 3 experiments), **P≤0.01 compared to GM at each concentration. (**B**) After removal of cell debris and whole cells, nsph-CM was concentrated 2500-fold and size fractionated into two parts; proteins >30 kDa (fraction A) and <30 kDa (fraction B). Fraction B was further concentrated 2500-fold as described in the Materials and Methods section. (**C**) Cells were plated at 2×10^3^ cells/ml in GM, nsph-CM, fraction A or fraction B. Nsph-CM, fractions A and B were reconstituted into GM to the original concentration prior to cell culture. Data are presented as mean ± SEM with *n*≥6 (from 3 experiments); *P≤0.05, **P≤0.01 compared to GM. (**D**) Fractions A and B derived from GM and nsph-CM were digested with trypsin and the mass of peptides evaluated using liquid chromatography-mass spectrometry (LCMS). The table shows peptide sequences unique to nsph-CM leading to the identification of DSD-proteoglycan, apolipoprotein E and cystatin C.

The standard culture condition for nsphs includes the B-27 supplement, bFGF and EGF. In such rich culture media it is difficult to study the effect of cell secreted factors by mass spectrometry mainly because of protein complexes formed in the presence of BSA. Thus we utilized a minimal media containing the N2 supplement and EGF alone. Under such condition we observed that nsph-CM stimulated nsph formation at various cell densities from clonal (10 cells/ml, equals 1 cell/well) to 1×10^4^ cells/ml (Fig. 1A). We fractionated the nsph-CM into fraction A (>30 kDa) and fraction B (<30 kDa; Fig. 1B) and found that in low density cultures fraction A has nsph stimulatory activity approximating that of whole nsph-CM, and fraction B stimulated nsph formation by 1.5-fold (Fig. 1C). The concentrated fractions A and B of nsph-CM were compared with the appropriate fractions of the growth medium (GM) by mass spectrometry. DSD-1-proteoglycan (also known as phosphacan), apolipoprotein E (ApoE) and cystatin C were identified as unique factors present in the nsph-CM (Fig. 1D).

### CSPG and ApoE is responsible for the nsph stimulatory effect of nsph-CM

**Figure 2:**
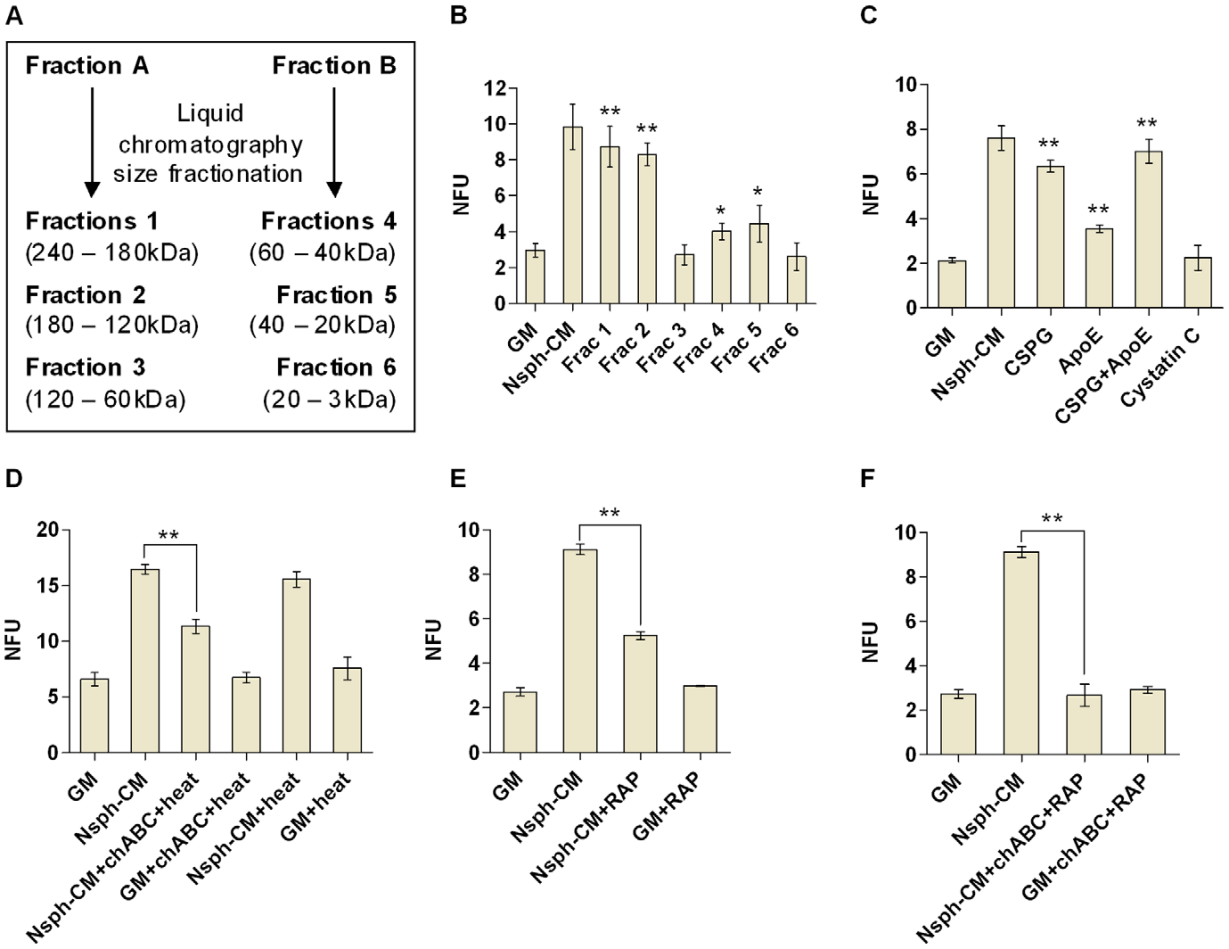
CSPG and ApoE are the components of nsph-CM responsible for stimulation of nsph formation. (**A**) Fractions A and B of nsph-CM were further fractionated into six fractions by size using liquid chromatography as shown. Cells were plated at 2×10^3^ cells/ml and NFUs measured after five days of culture as described in figure 1 with the following variations; (**B**) Addition of GM, nsph-CM or sub-fractions 1–6. Nsph-CM and sub-fractions 1–6 were reconstituted into GM to the original concentration prior to cell culture. (**C**) Addition of GM, nsph-CM, CSPG (50 µg/ml), ApoE (50 nM) or cystatin C (20 ng/ml). (**D**) Addition of GM and nsph-CM alone with and without heat inactivation, or GM and nsph-CM pre-digested with chABC (50 mU/ml) and heat inactivated. (**E**) Addition of GM and nsph-CM with and without recombinant receptor associated protein (RAP; 5 µM). (**F**) Addition of GM, nsph-CM, with and without chABC (50 mU/ml) treatment (including heat inactivation, see Materials and Methods) and RAP (5 µM). Data are presented as mean ± SEM with *n* = 4–11 (from 2–4 experiments); *P≤0.05, **P≤0.01 compared to GM or as indicated on the graphs.

To determine which of the identified proteins is likely to contribute to the nsph stimulatory effect of nsph-CM, we further fractionated fractions A and B (Fig. 2A). Fraction A was fractionated into sub-fractions 1 (240–180 kDa), 2 (180–120 kDa) and 3 (120–60 kDa). Sub-fractions 1 and 2 displayed nsph stimulatory activity similar to whole nsph-CM whereas sub-fraction 3 did not stimulate nsph formation (Fig. 2B). Fraction B was fractionated into sub-fractions 4 (60–40 kDa), 5 (40–20 kDa) and 6 (20–3 kDa). Sub-fractions 4 and 5 have similar nsph stimulatory activity as fraction B whereas sub-fraction 6 had no nsph stimulatory effect (Fig. 2B). This suggests that the stimulatory proteins are between 120–240 kDa and 20–60 kDa. From our list of identified proteins, DSD-1-proteoglycan is a CSPG with a calculated size of 173 kDa, ApoE is approximately 33 kDa, and cystatin C is approximately 13 kDa. Thus CSPG and ApoE are potential candidates responsible for the nsph-CM stimulation of nsph formation. To test our hypothesis, exogenous CSPG, ApoE and cystatin C were added to cells in GM. Indeed we found that exogenous CSPG and ApoE individually can recapitulate the effects of fractions A and B of nsph-CM respectively, and together reproduced the effect of the whole nsph-CM (Fig. 2C). Exogenous cystatin C did not stimulate nsph formation as expected, so this protein was not evaluated further. Cystatin C did however increase nsph size (NSC/NP proliferation; data not shown). To further confirm the role of CSPG, the nsph-CM was treated with chABC to digest the CS-GAGs, followed by heat inactivation of the enzyme. This chABC treatment resulted in a 51% reduction of the stimulatory effect of nsph-CM (Fig. 2D). Similar chABC treatment of GM did not affect nsph formation. Heating alone also did not compromise the stimulatory effect of nsph-CM. Thus, the reduction in the stimulatory effect of nsph-CM is due to chABC digestion of CSPGs in the nsph-CM, and not to the enzyme acting directly on the cells or heat inactivation of the nsph-CM. To confirm the role of ApoE we used the receptor-associated protein (RAP) to block ApoE binding to its receptor. This reduced the nsph stimulatory effect of nsph-CM by 60% (Fig. 2E). When both CSPG and ApoE were inhibited, the nsph stimulatory effect of nsph-CM was completely abolished (Fig. 2F). Thus both CSPG and ApoE are responsible for the nsph stimulatory effect of nsph-CM. In this study we focus on the role of CSPG in NSC survival and growth, while the role of ApoE was investigated elsewhere (submitted).

### CSPG is essential for nsph formation and proliferation

**Figure 3:**
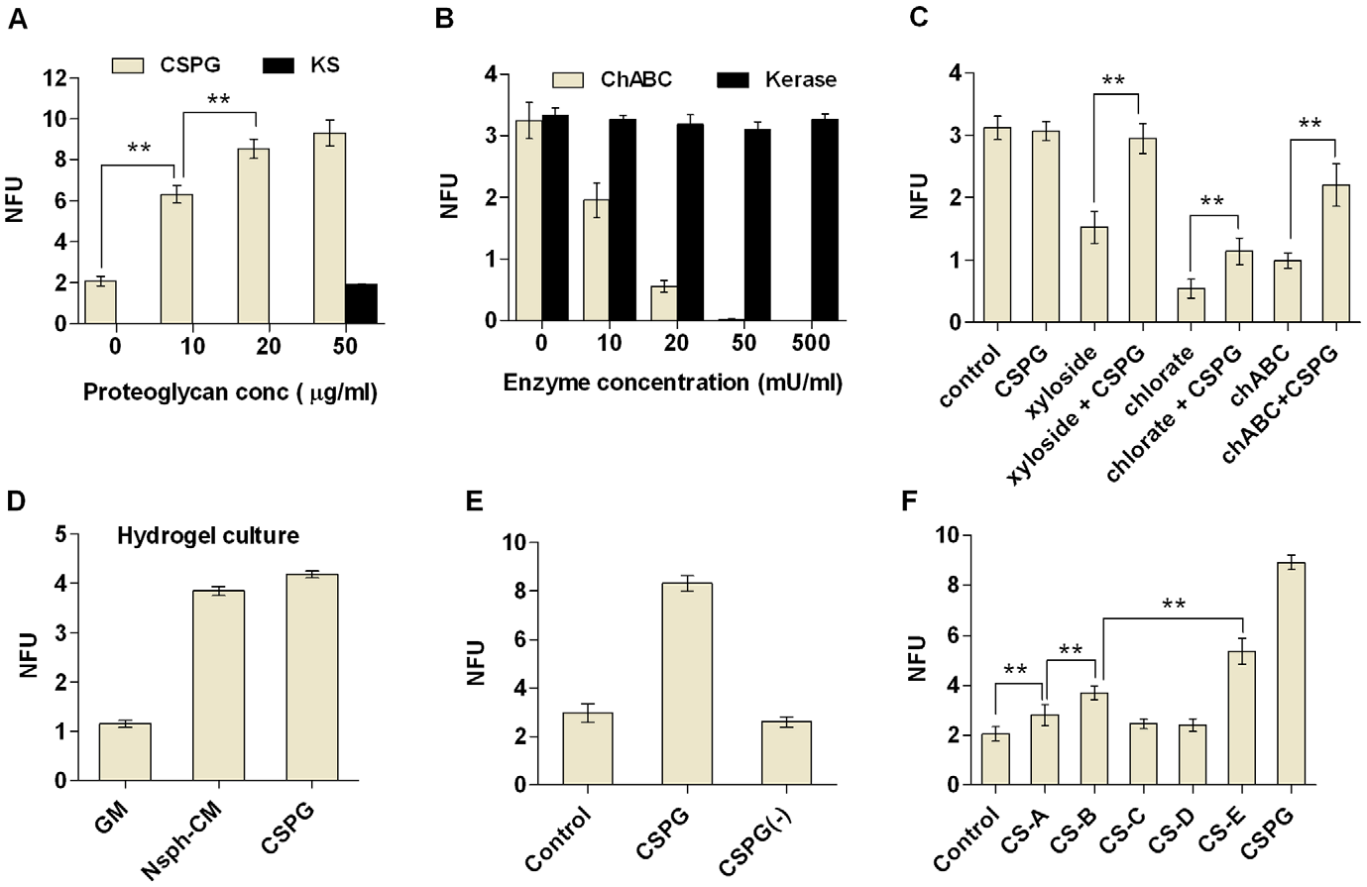
Specificity of CSPG mediated stimulation of nsph formation. Cells were plated at 2×10^3^ cells/ml and NFUs measured after five days of culture as described in figure 1 (except D). Data shown in the bar charts (**A–F**) examine the effect of the following on NFUs; (**A**) CSPG (10–50 µg/ml) and keratin sulfate (KS; 50 µg/ml). (**B**) ChABC (10–50 mU/ml) and keratinase (Kerase; 10–500 mU/ml). (**C**) GM, CSPG (50 µg/ml); xyloside (150 µM), chlorate (50 mM) and chABC (50 mU/ml) with and without CSPG (50 µg/ml). (**D**) The effect of GM, nsph-CM and CSPG (50 µg/ml) on NFUs measured in hydrogel. Cells were plated at 2.5×10^3^ cells/ml in hydrogel medium (see Material & Methods) and NFU measured after five days. (**E**) Nsphs grown in the presence of CSPG were dissociated and replated at 2×10^3^ cells/ml with and without CSPG (50 µg/ml). Secondary nsph formation was evaluated after five days. (**F**) GM, CSPG (50 µg/ml), chondroitin sulfate A–E (CS-A to CS-E; all at 50 µg/ml). Data are presented as mean ± SEM with *n* = 3–12 (from 2–4 experiments); ** *P*≤0.01.

To determine whether the increase in nsph formation is specific to CSPG, we tested both exogenous addition of proteoglycans and inhibition of endogenous proteoglycans. Exogenous CSPG was able to stimulate nsph formation in a dose-dependent manner whereas another proteoglycan, keratan sulfate (KS), had no effect (Fig. 3A). Digesting the CS-GAG chains with chABC resulted in a dose-dependent reduction in nsph formation (Fig. 3B), whereas digestion of KS-GAG chains with keratanase did not affect nsph formation even when used at 10 times the maximum concentration of chABC. This supports the idea that nsph formation specifically requires CSPG. The chABC treatment not only inhibited nsph formation but also disrupted the 3D structure of any nsphs that formed (Fig. S1). To ensure that the reduction in nsph formation is not due to non-specific interference of the nsph structure, we also disrupted CSPG biosynthesis with sodium chlorate and β-D-xyloside. Both compounds were able to inhibit nsph formation similar to chABC but without any disruption to the nsph structure (Fig. 3C). Furthermore, addition of CSPG was able to partially rescue the inhibitory effect of all three CSPG inhibitors (Fig. 3C). The rescue effect in the presence of chABC is likely due to exogenous CSPG saturating the enzyme added.

In free-floating cultures cell aggregation can occur [18], [22]. This can promote nsph formation in cells that may not have intrinsic nsph forming abilities, complicating the analysis. To test whether CSPG can stimulate nsph formation under non-aggregating conditions, a hydrogel culturing system was used to immobilize cells. Similar to low density suspension cultures, both nsph-CM and CSPG stimulated nsph formation in hydrogel cultures, and their effects were comparable (Fig. 3D). Thus both nsph-CM and CSPG directly stimulate nsph formation in the absence of cell aggregation. The stimulatory effect of CSPG was temporary. When CSPG-generated nsphs were dissociated and replated without CSPG, nsph formation rate returned to control levels (Fig. 3E). CSPGs are known to function mainly through their GAG chains. Indeed we found that the GAGs CS-A, B and E can promote nsph formation whereas CS-C and -D had no effect (Fig. 3F). Images of all cell culture conditions are presented in figure S1.

In addition to stimulating nsph formation, exogenous CSPG increased total cell number (Fig. S2A) and nsph size (Fig. S2D). The increase in nsph size was observed in both suspension cultures and immobilized hydrogel cultures. Thus CSPG increases nsph size as a result of increased proliferation rather than aggregation. Inhibiting endogenous CSPG with chABC, chlorate and xyloside also reduced cell numbers (Fig. S2B) and nsph size (Fig. S2E). The ability of chABC to break down the 3D structure of the nsphs suggests that CSPG is involved in maintaining the nsph structure. Endogenous CSPG may regulate cell proliferation as a result of its structural function, i.e. holding the cells in close contact allows cell-cell signaling to stimulate proliferation. To investigate this possibility we switched to an adherent monolayer culture system. Again, chABC, chlorate and xyloside treatment inhibited NSC/NP proliferation, while CSPG increased proliferation (Fig. S2C). Thus CSPG is involved in NSC/NP proliferation independently of its nsph structural role. Analysis of cell population doubling time show that CSPG significantly reduces the population doubling time of low density nsph assays (NSA) and adherent cultures, while CSPG inhibitors increased the doubling times in all culture conditions (Fig. S2F).

### CSPG stimulates NSC survival

**Figure 4:**
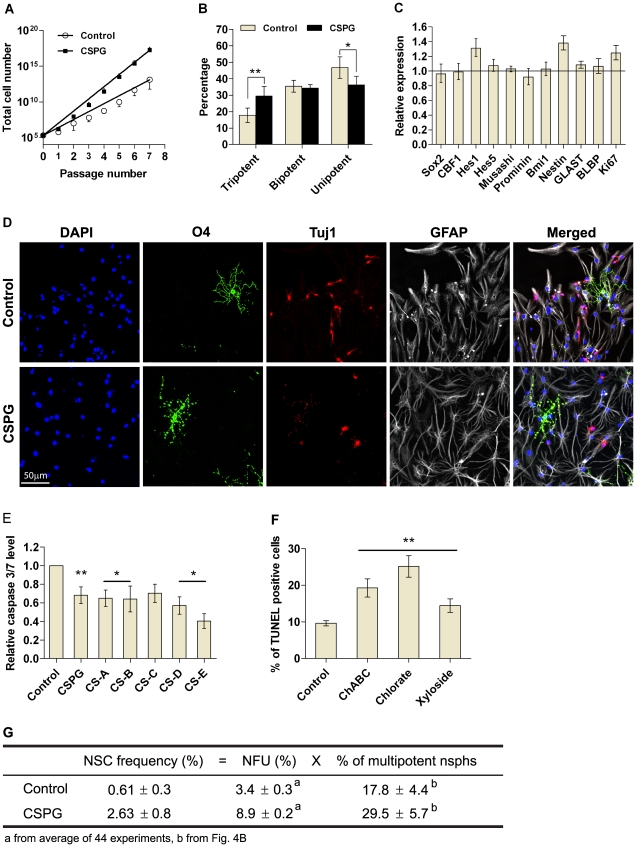
CSPG stimulates NSC survival. (**A**) Cells were plated at 2×10^3^ cells/ml with CSPG (50 µg/ml) or without (control). Nsphs were passaged every seven days with cells replated at the same density and treatment conditions. Cell numbers were counted at each passage and plotted against passage number. (**B**) Statistical analysis of the multipotency of individual nsphs. Individual clonal nsphs grown with or without CSPG (50 µg/ml) were differentiated (see Materials and Methods) and scored as follows; tripotent (neurons, astrocytes and oligodendrocytes), bipotent (neurons/astrocytes or oligodendrocytes/astrocytes), unipotent (astrocytes). Data are presented as mean ± SEM with *n*≥347 (from 6 experiments); * *P*≤0.05, ** *P*≤0.01. (**C**) Gene expression of individual clonal nsphs grown with or without CSPG (50 µg/ml) was measured by quantitative RT-PCR as described in the supplementary Materials and Methods S1. The bar chart show changes in gene expression induced by CSPG with a value of 1 indicating no change. Data are presented as mean ±95% confidence interval with *n*≥70 (from 3–10 experiments). (**D**) Representative images of differentiated nsphs stained for the lineage markers O4 (oligodendrocyte; green), Tuj1 (neuron, red) and GFAP (astrocyte, gray). Nuclei were counterstained with DAPI (blue). The merged image shows co-localization of all three cell types. Scale bar  = 50 µm at 20X objective. (**E**) Dissociated cells were plated at bulk density in GM with or without CSPG or CS-A to –E (all at 50 µg/ml) for 24 hours. Bar chart shows active caspase 3/7 level as determined by the Caspase-Glo 3/7 kit. (**F**) Dissociated cells were plated onto PLL coated coverslips at 5×10^5^ cells/13 mm coverslip in GM containing chABC (20 mU/ml), chlorate (50 mM) or xyloside (150 µM) for 24 hours. Cells were fixed and stained with DeadEnd Fluorometric TUNEL kit. Bar chart shows percentage of TUNEL positive cells with each treatment. Data are presented as means ± SEM with *n*≥9 (from 4–5 experiments); * *P*≤0.05, ** *P*≤0.01 compared to control, bar represent same significance level compared to control. (**G**) NSC frequency was calculated by multiplying NFUs with the percentage of multipotent nsphs.

Both NSCs and NPs can form nsphs [23]. To determine whether CSPG was affecting NSC survival it was critical to assess CSPG-generated nsphs for their self-renewal and multipotent characteristics. CSPG-treated cells can be passaged for at least seven passages similar to control cells, but gave rise to more cells (Fig. 4A). To score for multipotency, we differentiated individual nsphs generated from clonal low density cultures. Upon differentiation, 17.8±4.4% of control nsphs were found to be multipotent, producing neurons, oligodendrocytes and astrocytes (Fig. 4B & D). CSPG treatment significantly increased the percentage of tripotent nsphs by 11.7%, and decreased the percentage of unipotent (astrocyte only) nsphs by 10.6% as compared to the control. The percentage of bipotent nsphs, neurons/astrocytes or oligodendrocytes/astrocytes, were unaffected (Fig. 4B). Astrocytes were always present, thus there were no neuron/oligodendrocyte bipotent nsphs.

We further characterized the nature of CSPG-generated nsphs by gene profiling individual nsphs and using the neural colony-forming cell assay (NCFCA). A panel of genes related to NSCs remained largely unchanged. Significant increases were observed for Hes1, Nestin and Ki67 (Fig. 4C). The NCFCA is based on the hypothesis that NSCs have greater proliferative potential and will give rise to larger colonies than progenitors [24]. CSPG-treated cells generated fewer colonies between 0.5 and 0.8 mm in diameter, with a significant increase in colonies between 0.8 and 1.2 mm as compared to the control (Fig. S3A). Conversely chABC, chlorate and xyloside treatment increased the percentage of colonies smaller than 0.5 mm and decreased percentage of colonies in the 0.5–1 mm category (Fig. S3B). Taken together, these observations on self-renewal, multipotency, gene expression and colony formation suggest that CSPG increases NSC survival. In support of this survival role, cells incubated with CSPG or CS-A to -E for 24 hours showed significantly less active caspase 3/7 as compared to untreated cells (Fig. 4E). Conversely, chABC, chlorate and xyloside treatments for 24 hours significantly increased the percentage of TUNEL positive cells (Fig. 4F). To evaluate the effect of CSPG on NSC survival, we calculated the NSC frequency as a product of clonal NFU and multipotent nsph percentage. CSPG treatment increased the NSC frequency by more than four-fold (Fig. 4G).

### CSPG stimulates ESC-derived nsph formation

**Figure 5:**
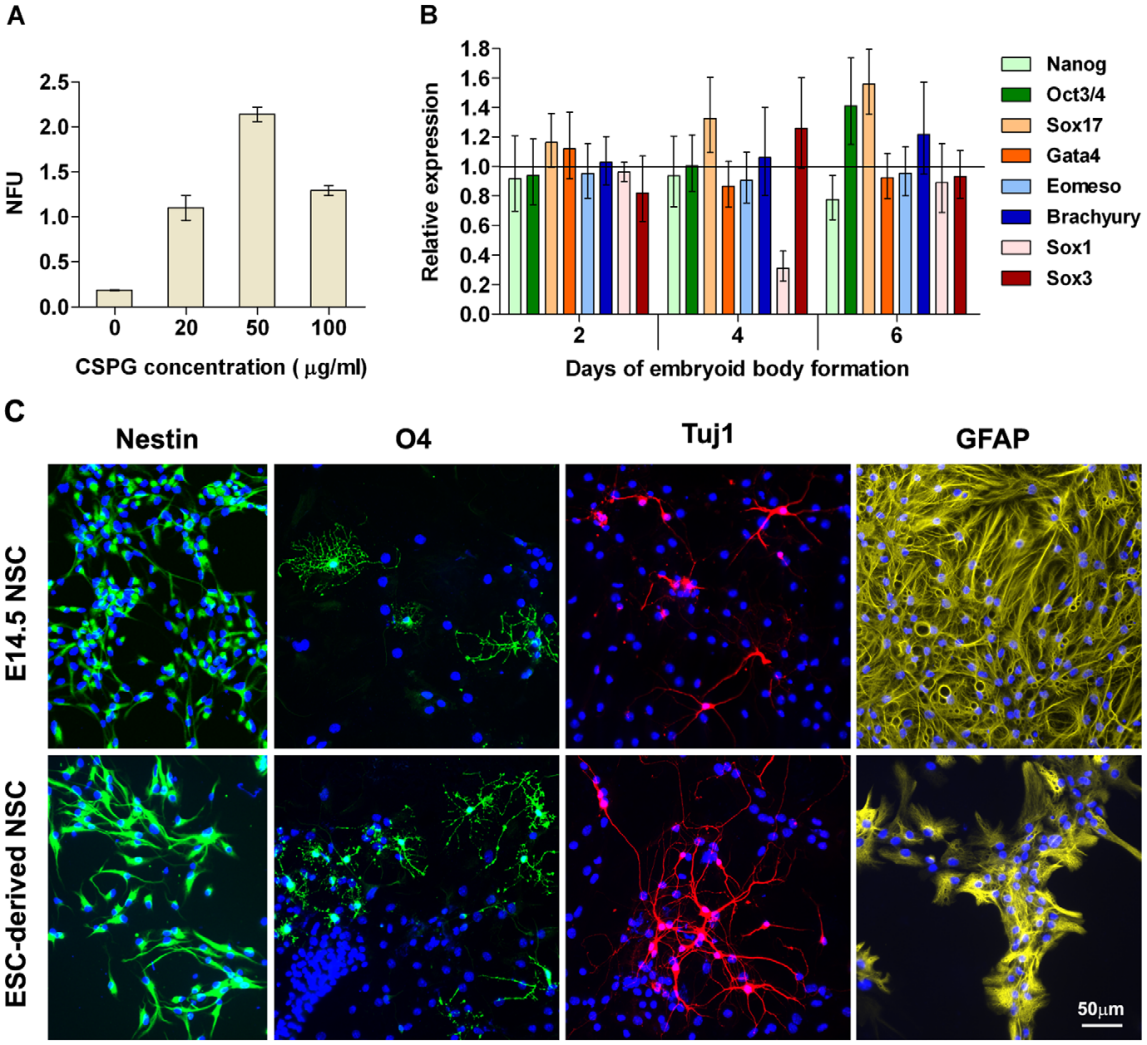
CSPG stimulates nsph formation from ESC. (**A**) ESC cells were cultured at 10^5^ cells/ml with or without CSPG (20–100 µg/ml) in nsph growth medium and NFUs measured after 21 days. Data are presented as mean ± SEM with *n*≥3 (from 1 representative experiment). Similar results were obtained in two other experiments. (**B**) Expression analysis of pluripotency genes (Nanog and Oct3/4), endoderm genes (Sox17 and Gata4), mesoderm genes (Eomeso and Brachyury) and ectoderm genes (Sox1 and Sox3) was measured by quantitative RT-PCR as described in the supplementary Materials and Methods S1. The bar chart shows CSPG (50 µg/ml) induced changes in gene expression relative to control with a value of 1 indicating no change. Data are presented as mean ± 95% confidence interval with *n* = 8 (from 4 experiments). (**C**) Undifferentiated ESC-derived nsphs and E14.5 nsphs were stained for nestin. Both types of nsphs were dissociated for differentiation and stained for lineage markers as described in figure 4 and the supplementary Materials and Methods S1. Scale bar  = 50 µm at 20X objective.

ESCs can spontaneously form nsphs at a very low frequency when cultured in NSC growth medium. We next asked whether CSPG also affects ESC-derived nsphs. Addition of exogenous CSPG increased the percentage of ESC-derived nsphs (Fig 5A). The CSPG-generated ESC-derived NSCs were more than 90% nestin positive (n = 5) and can differentiate into neurons, oligodendrocytes and astrocytes (Fig 5C). To determine whether CSPG increased ES-derived nsph formation by directed differentiation or by expanding the pool of cells already committed to the neural lineage, we assessed the effects of CSPG on ESCs cultured in ESC medium without LIF. Under this culture condition, ESCs differentiate randomly into cells of the three germ layers as indicated by the expression of endoderm genes (Sox17 and Gata4), mesoderm genes (Eomeso and Brachyury) and ectoderm genes (Sox1 and Sox3; data not shown). CSPG treatment did not alter this differentiation process since no persistent changes in the expression of these genetic markers were observed (Fig. 5B). The expression level of pluripotent genes (Nanog and Oct3/4) was also not significantly altered. This suggests that CSPG did not induce differentiation of ESCs into NSCs but possibly promoted survival and proliferation of committed NSCs/NPs.

### CSPG stimulates nsph formation via enhancement of EGFR, JAK and PI3K signaling

**Figure 6:**
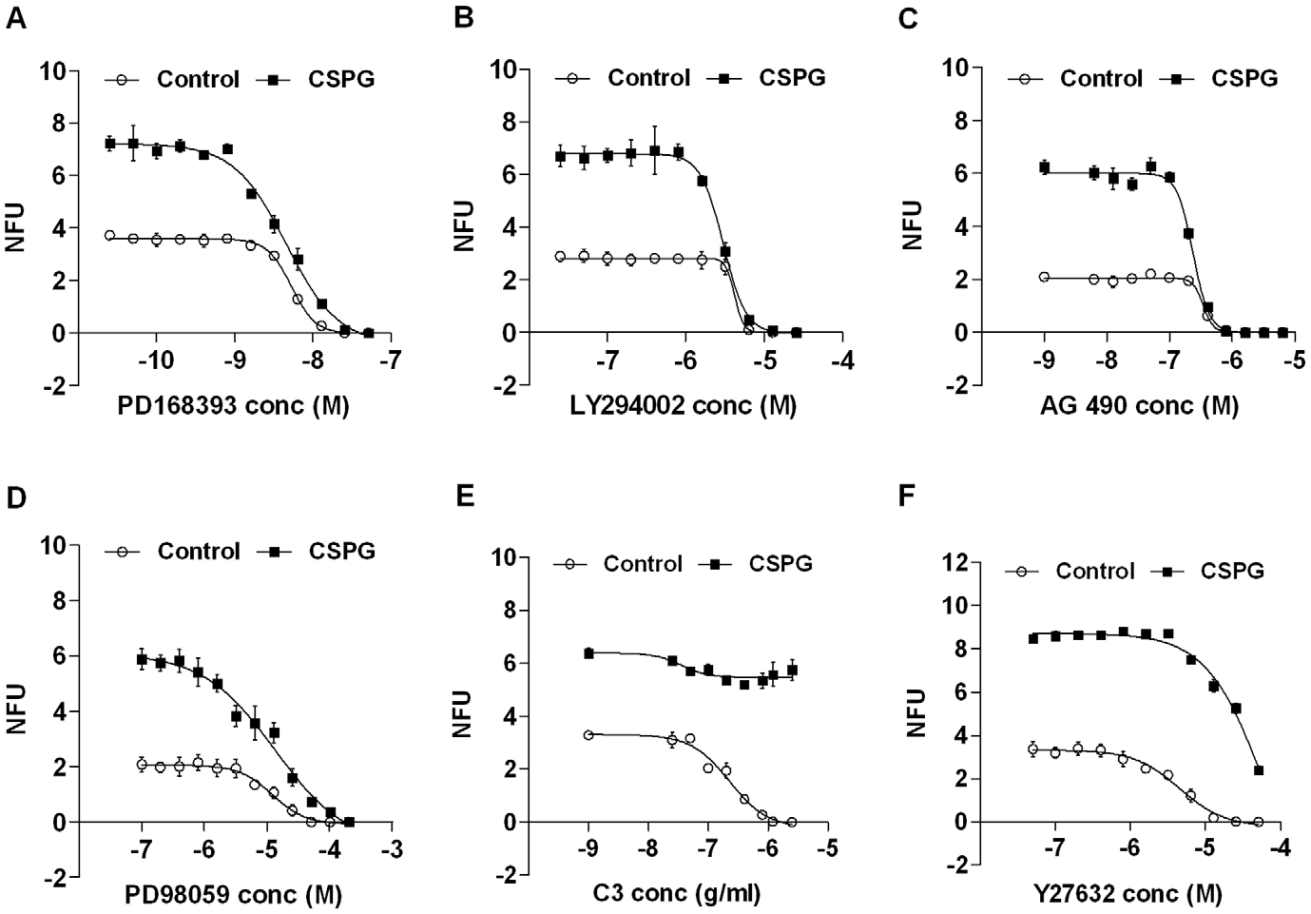
Effect of inhibitors of EGFR and RhoA signaling pathways on NFUs. Cells were grown at 2×10^3^ cells/ml, inhibitors added at the indicated concentrations and nsph formation followed for five days in the presence or absence of CSPG (50 µg/ml). (**A**) EGFR kinase inhibitor PD168393 concentration range (50 pM–50 nM). (**B**) PI3K inhibitor LY294002 concentration range (25 nM–25 µM). (**C**) JAK inhibitor AG490 concentration range (6.25 nM–6.4 µM). (**D**) ERK inhibitor PD98059 concentration range (100 nM–200 µM). (**E**) RhoA inhibitor C3 concentration range (25 ng/ml–25 µg/ml). (**F**) Rho kinase inhibitor Y27632 concentration range (50 nM–50 µM). Data are presented as mean ± SEM, *n* = 3 from 1 representative experiment. Similar results were obtained in two other experiments.

To begin to understand how CSPG may signal within NSCs, we analyzed the EGFR and Rho pathways. The EGFR pathway is essential for NSC survival and proliferation while CSPG is known to signal via Rho/ROCK [25]. Since nsph formation is a long-term process, we used inhibitors of EGFR and Rho/ROCK signaling to determine the effect on nsph formation in the presence of CSPG. Inhibition of EGFR kinase activity with PD168393 reduced nsph formation in both control and CSPG treated cultures (Fig. 6A). The IC50 values for nsph formation (IC50_NF_) of PD168393 for control and CSPG-treated cells are 5.87±1.7 nM and 2.83±0.89 nM respectively (means ± SEM, *P*≤0.05). Thus the concentration of PD168393 required to reduce 50% of nsph formation is significantly less for CSPG-treated cells than control, indicating that CSPG stimulation of nsph formation may be preferentially inhibited over basal nsph formation. Stimulation of EGFR leads to the activation of PI3K, JAK and ERK. Inhibition of PI3K with LY294002 significantly reduced CSPG-stimulated nsph formation (Fig. 6B). The IC50_NF_ of LY294002 for control and CPSG-treated cells are 3.78±0.4 µM and 2.97±0.23 µM respectively (means ± SEM, *P*≤0.05). Inhibition of JAK with AG490 also reduced CSPG-stimulated nsph formation (Fig. 6C). The IC50_NF_ of AG490 for control and CSPG-generated cells are 0.4±0.069 µM and 0.33±0.064 µM respectively (means ± SEM, *P*≤0.05). Conversely, inhibition of ERK with PD98059 (Fig. 6D) gave similar IC50_NF_ values for control and CPSG-treated cells, 12.4±0.18 µM and 12.8±0.69 µM respectively. This indicates that PD98059 is inhibiting control and CSPG-treated cells at the same rate. Inhibition of Rho GTPases with C3 transferase was able to inhibit nsph formation in control cultures. However, in the presence of CSPG, C3 had minimal effect on nsph formation (Fig. 6E). The Rho kinase inhibitor Y27632 was also unable to abolish CSPG stimulation of nsph formation (Fig. 6F). The IC50_NF_ of Y27632 for control and CSPG-generated cells are 4.24±0.68 µM and 59.6±2 µM respectively (means ± SEM, *P*≤0.001).

Further analysis of the inhibitor data show that the concentration of LY294002, AG490 and PD168393 that abolished the CSPG stimulatory effect only inhibited nsph formation in control cultures by 10, 20 and 30%, respectively (Fig. S4). In contrast, the concentration of PD98059 that abolished the CSPG stimulatory effect reduced nsph formation in control cultures by 79%. While for Y27632, no nsph formation occurred at 50 µM, the concentration required to inhibit the CSPG stimulatory effect. This suggests that CSPG preferentially signals through PI3K, JAK and EGFR.

### CSPG stimulates EGFR signaling pathways

**Figure 7:**
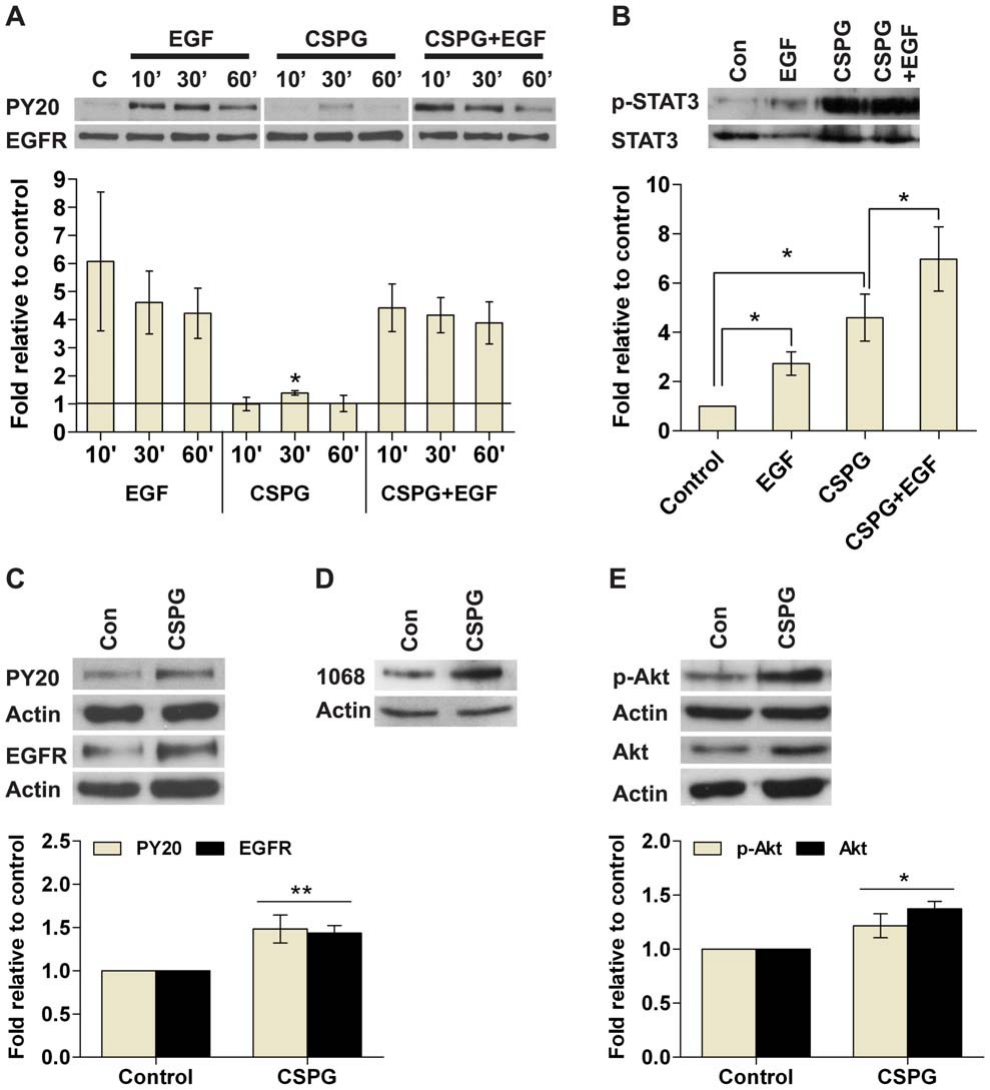
CSPG stimulates EGFR signaling pathways. Kinase activity was estimated by using Western analysis. Whole cell lysates were separated by SDS-PAGE and probed with phosphotyrosine antibodies; PY20, p-Akt, p-EGFR (1068), and p-STAT3. The respective total protein signal was used to normalize protein loading. For each figure, the images are representative Western blots while the bar charts below show quantification of signals expressed relative to control. Data are presented as mean ± SEM with *n*≥4 (from ≥3 experiments); * *P*≤0.05, ** *P*≤0.01 compared to control (con) or as indicated on the graphs. (**A**) Nsphs were dissociated into single cells and cultured in growth medium without EGF for three hours followed by stimulation with EGF (20 ng/ml), CSPG (50 µg/ml) or both for 10 to 60 min. EGFR activation status as seen by PY20. (**B**) Cells were cultured as in (A) followed by stimulation with EGF (20 ng/ml) for 30 min or CSPG (50 µg/ml) for three hours. Cells pretreated with CSPG (50 µg/ml) for three hours were stimulated with EGF (20 ng/ml) for 30 min (CSPG + EGF). STAT3 activation status as seen by p-STAT3. (**C**) Cells were cultured for six days in the presence of EGF (20 ng/ml), with or without CSPG (50 µg/ml). EGFR activation status as seen by PY20 and EGFR levels. (**D**) Cells were cultured as in (C) and EGFR phosphorylation at tyrosine residue 1068 was evaluated using the p-EGFR (1068) antibody. (**E**) Cells were cultured as in (C) and Akt activation status as seen by p-Akt and Akt levels.

Next we use Western analysis to determine whether CSPG can directly stimulate the EGFR signaling pathways. CSPG directly stimulated EGFR phosphorylation (Fig. 7A) after 30 minutes (1.4±0.08 fold relative to control, *P*≤0.05). However, CSPG did not enhance EGF stimulation of EGFR. Although there is a trend for reduced EGFR phosphorylation at the 60 minutes time point with CSPG and EGF stimulation compared to EGF alone, the difference is not significant when results from multiple experiments were combined as the error bars were large. Specific phospho-EGFR antibodies revealed that CSPG stimulates EGFR phosphorylation at tyrosine 1068 (Fig. 7D). CSPG stimulated STAT3, a downstream effector of JAK, within three hours (Fig. 7B). EGF alone also stimulated STAT3 phosphorylation as expected. In cells pretreated with CSPG, EGF induced further stimulation of STAT3 which approximated the sum of the individual stimulants alone. This result is significant when multiple experiments were combined (*P*<0.05). After six days in culture, CSPG-generated cells showed a significant increase in the levels of EGFR and phospho-EGFR (Fig. 7C), as well as an increase in the levels of Akt and phospho-Akt, a downstream effector of PI3K (Fig. 7E). CSPG did not directly stimulate Akt phosphorylation in short term experiments, nor affected ERK and phospho-ERK levels in either short or long term cultures (data not shown). Together these results suggest that CSPG can directly stimulate phosphorylation of EGFR and STAT3, as well as upregulate the long term expression of EGFR and Akt.

## Discussion

The present work began with the observation that mouse E14.5 nsph-CM stimulates nsph formation. Following mass spectrometry identification of CSPG, ApoE and cystatin C, we showed by both inhibition of endogenous protein and reconstitution with exogenous protein that CSPG and ApoE can fully account for the nsph stimulatory effect of nsph-CM. Although we did not inhibit cystatin C in the nsph-CM, we reasoned that cystatin C is unlikely to play a stimulatory role since neither the nsph-CM fraction that is likely to contain cystatin C (sub-fraction 6), nor reconstitution with exogenous cystatin C were able to stimulate nsph formation. Previously, cystatin C was isolated from adult rat hippocampal progenitor-CM and demonstrated to stimulate NSC/NP proliferation and clone formation [3]. A possible reason for the differences in our data could be the embryonic NSCs/NPs that we use do not require cystatin C for nsph formation whereas this protein is more essential for adult NSCs/NPs. It is well known that NSCs/NPs alter their responses to growth factors over time [26].

To confirm the involvement of CSPG we showed that addition of pure CSPG can recapitulate the effect of nsph-CM and stimulates nsph formation and proliferation under clonal conditions. On the other hand, digestion of CSPG with chABC inhibited nsph formation. A recent publication showed that chABC inhibits FGF-dependent but not EGF-dependent nsph formation [27]. We find this difficult to reconcile since our cells are cultured in EGF only media and we observe a dose-dependent inhibition of nsph formation with chABC. We can only speculate that this may arise from experimental differences.

We found that the effects on nsph formation are specific to CSPG since neither exogenous addition of KS-GAG nor disruption of endogenous KS-GAG affected nsph formation. Interestingly inhibition of CSPG with chABC not only reduces nsph formation but also disrupts the integrity of the nsph structure. CSPG is thought to function through its CS-GAGs to form a major component of the perineuronal net (PNN), a specialized ECM in the CNS which is involved in both synaptic and structural plasticity of the brain [28]. Furthermore, intraventricular injection of chABC disrupts the organization of the embryonic ventricular zone [17]. Thus CS-GAG chains are likely to be crucial for maintaining the structure of nsphs *in vitro* and the neurogenic zone *in vivo*. Indeed, we found that the CS-GAGs alone can stimulate nsph formation. Previously, CS-B, -D and -E units have been shown to promote FGF-2-mediated proliferation of rat embryonic NSCs/NPs [29]. Here, we show that CS-A, -B and -E stimulates nsph formation in EGF-dependent mouse embryonic NSCs/NPs, whereas CS-C and -D does not. Thus CSPG can modulate nsph formation using different sulfation motifs.

### CSPG stimulates NSC survival

One of the key questions that have not been addressed is the role of cell-secreted CSPGs in NSC/NP survival. The defining features of an NSC include self-renewal and multipotency. *In vitro*, self-renewal is often measured by the ability of NSCs to generate secondary nsphs. However, nsph formation is not a property restricted to NSCs as NPs can also form nsphs [30]. As for evaluation of multipotency, when differentiation is performed using dissociated cells, or a pool of nsphs, it is not possible to determine whether the multiple cell types obtained came from tripotent, bipotent or unipotent nsphs. The first type of nsphs will suggest the presence of NSCs whereas the later two indicates the presence of NPs. Thus CSPG-generated nsphs were evaluated in terms of; (a) the ability to survive long-term passage, since progenitor-derived nsphs have limited self-renewal capacity [31], and (b) the ability of individual clonal nsphs to give rise to different lineages. We showed that CSPG-generated cells can be serially passaged for at least seven passages, thus fulfilling the NSC criterion of extensive self-renewal. Differentiation of individual clonal nsphs showed that CSPG treatment increases the percentage of tripotent nsphs. Together these data indicate that CSPG specifically increases NSC survival. In support of this survival role both CSPG and CS-GAGs decreased active caspase 3/7 level. Using the NCFCA we showed that CSPG treatment did not alter the percentage of >2 mm colonies (putative stem cell-derived colonies [24]). However, CSPG stimulated nsphs did form larger colonies (0.8-1.2 mm) compared to untreated neural progenitors. The lack of changes in the >2 mm colonies is likely due to the low density and minimal medium culturing system that we use compared to the high density and enriched medium cultures as recommended by the original authors [24]. Nevertheless, our data indicates greater proliferation upon CSPG stimulation and is consistent with CSPG increasing NSC frequency. The nsph stimulatory effect of CSPG is transient. When CSPG-generated nsphs were dissociated and replated without CSPG, nsph formation rate returned to control levels. This suggests that CSPG acts as a survival factor for existing NSCs rather than promotes NSC self-renewal.

The NSA has been widely used to study NSCs. But when used alone, it cannot enumerate the NSC frequency. The enumeration of NSC frequency requires demonstration of clonality and multipotency in the same nsph. Using clonal nsphs we have demonstrated the presence of multipotent nsphs. Based on these data, we estimated that the NSC frequency is 0.61±0.3% and CSPG increases this frequency by more than four-fold (Fig. 4E), indicating that CSPG is increasing NSC survival.

Previously cystatin C, a factor present in the nsph-CM, have been shown to stimulate NSC formation from ESCs [4]–[5]. Here we show that in addition to its effect on NSCs, CSPG also stimulates nsph formation from ESCs. CSPG does not stimulate the differentiation of ESCs, thus it is likely to enhance the survival of ESC-derived NSCs similar to its effect on brain-derived NSCs. Given the low rate at which ESCs normally convert to nsphs (0.2%), addition of CSPG represents a useful tool to generate ESC-derived NSCs, and will assist in delineating the developmental processes involved in the transition from ESC to NSC.

### CSPG stimulates nsph formation via enhancement of EGFR, JAK/STAT3 and PI3K/Akt signaling

To determine which signaling pathway(s) may be involved in CSPG's effect on NSC survival we carried out both short and long term assays. The EGFR and Rho signaling pathways were selected since EGF is known to be required for nsph propagation and CSPG signals via RhoA in neurons. The inhibitor studies suggest EGFR, JAK and PI3K are the most likely proteins through which CSPG signals, since the stimulatory effect of CSPG can be abolished with inhibitors of these pathways at concentrations that had minimal effect on control cultures. Reduced IC50_NF_ values were also observed for CSPG cultures. In contrast, inhibition of MEK, RhoA and ROCK either did not affect CSPG stimulation (RhoA) or inhibits CSPG stimulation at concentrations that produced near-complete or complete inhibition of nsph formation in control cultures (MEK and ROCK respectively). This suggests that CSPG is unlikely to signal via MEK, RhoA and ROCK. The inhibitor studies are supported by the observations that CSPG can directly stimulate EGFR and STAT3 phosphorylation, as well as regulate long-term expression of EGFR and Akt. Since the direct stimulation of EGFR phosphorylation is minimal and not apparent in the presence of EGF it is likely that the long-term upregulation of EGFR expression is more important for CSPG signaling. Similarly CSPG may signal via the PI3K/Akt pathway by long-term upregulation of Akt expression rather than directly stimulating this protein.

The EGFR and PI3K/Akt pathways are known to be involved in nsph formation and NSC/NP proliferation [32]–[34]. CSPG has also been shown to regulate EGFR [35]–[38], and PI3K/Akt [39]–[42] signaling independently in various cell types. However, the work presented here demonstrates that CSPG may enhance signaling of both proteins in NSCs. The JAK/STAT pathway has also been demonstrated in NSCs/NPs [43]–[44], and a recent article indicates that CS-A can stimulate STAT3/4 gene expression in splenocytes [45]. Our data suggest that this pathway, CSPG stimulation of STAT3, also occurs in NSCs. However, our data shows that a combination of CSPG and EGF produced greater stimulation of STAT3 than the individual stimulants. This suggests that CSPG may enhance STAT3 signaling via pathways besides EGFR. Cytokines activate the JAK/STAT pathway via the glycoprotein receptor gp130 [46]. This pathway is involved in neurogenesis and NSC self-renewal [47]–[48]. The gp130 receptor may be a potential route through which CSPG can stimulate JAK/STAT to promote NSC survival. More recently, the integrin pathway has also been shown to be involved in CSPG signaling in rat neural progenitor cells [49]. Thus CSPG may signal via multiple pathways to regulate neural progenitor growth and differentiation.

### Conclusion

CSPGs are involved in CNS injury and inhibition of regeneration [50]. The findings reported here of CSPG stimulation of NSC survival and proliferation suggest that CSPG could aid in brain repair. How can these distinct functions of CSPG be rationalized? One possible explanation is that the barrier property of CSPGs is involved in both inhibition of regeneration and maintenance of NSCs. CSPG is thought to inhibit neuroregeneration by regulating the availability of growth factors to the growing axons as well as through direct signaling pathways [51]. CSPGs may also form barriers around NSCs and regulate proliferation, differentiation and apoptotic signals to maintain the NSC state. Such a model has been proposed for CSPG maintenance of the articular cartilage stem cell niche [52]. We have shown here that CSPG is an essential component of the nsph-CM. It is involved in regulating NSC survival and proliferation, in nsph formation and maintenance, possibly via enhancement of EGFR, JAK/STAT3 and PI3K signaling pathways.

## Supporting Information

Materials and Methods S1 Material and methods for apoptosis assays, single nsph gene profiling, neural colony forming cell assay and dissociated cell differentiation.
(DOC)

Figure S1 **Images of cell culture systems.** Dissociated cells were cultured in suspension, in hydrogel or on poly-L-lysine coated dishes to form adherent cultures. For control and CSPG (50 µg/ml) treated cultures, cells were plated at 2×10^3^ cells/ml. CSPG treatment stimulated proliferation in all culture conditions. For chABC (20 mU/ml) treated cultures, cells were plated at 2×10^4^ cells/ml. ChABC brokedown the 3D nsph into loss cell clusters (arrow, left panel) and induced cell attachment and proliferation as an adherent layer (right panel). Arrow head in the left panel marks a normal nsph. Scale bar  = 100 µm at 10X objective.
(TIF)

Figure S2 **CSPG treatment increased NSC/NP proliferation.** Cells were cultured in suspension at 2×10^3^ cells/ml (**A**) and 2×10^4^ cells/ml (**B**) or as adherent culture (**C**; 1×10^4^ cells/ml). The treatment conditions were CSPG (50 µg/ml), chABC (50 mU/ml), sodium chlorate (20 mM) or xyloside (150 µM). Total viable cells were determined on alternate days with the CellTitre Glo assay kit (Promega) measuring ATP level in viable cells by bioluminescence signals. Graphs show luminescence level against days in vitro. (**D**) and (**E**) The diameter of nsphs grown with and without CSPG (50 µg/ml), chABC (50 mU/ml), sodium chlorate (20 mM) or xyloside (150 µM) in suspension culture were measured and divided into three categories, <50 µm, 50–100 µm and >100 µm. Graph show percentage of nsph in each size category. For (D) nsph diameter was also measured in hydrogel. (**F**) Population doubling time (hours) for cells cultured under different conditions calculated from the above experiments using the GraphPad Prism software. Data are presented as mean ± SEM with *n*≥6 (from 2–6 experiments); *P≤0.05, **P≤0.01 compared to the control in each size category for (D) and in each density for (F); a = *P*≤0.01 compared to control and b = *P*≤0.01 compared to inhibitor only cultures in (E); N.D., not determined; NS, not significant.
(TIF)

Figure S3 **Neural colony forming assay.** (**A**) Nsphs grown with or without CSPG (50 µg/ml) for seven days were dissociated and plated at 2.5×10^3^ cells/ml in NCFCA collagen medium. Colony number and size were recorded after three weeks. The bar chart shows the percentage of colonies above 0.5 mm in diameter. (**B**) Nsphs grown with or without chABC (50 mU/ml), sodium chlorate (20 mM) or xyloside (150 µM) for four days were analyzed as in (A). The bar chart shows the percentage of colonies in each size category. Data are presented as means ± SEM; *n*≥20 (from 5 experiments); * *P*≤0.05, ** *P*≤0.01 as compared to control, bar represent same significance level compared to control.
(TIF)

Figure S4 **Interpretation of chemical inhibition data.** (**A**) Diagram illustrating estimation of the inhibitor concentration that abolishes the stimulatory effect of CSPG (S) and its effect on control nsph formation rate. (**B**) Table summarizing the estimations from diagram (A) for each inhibitor as well as the IC50 values for nsph formation (IC50_NF_). Data are presented as means ± SEM with *n* = 9 (from 3 experiments); *P≤0.05, **P≤0.01 compared to control; NS, not significant.
(TIF)
