# Supplementary material for: CSPG Is a Secreted Factor that Stimulates Neural Stem Cell Survival Possibly by Enhanced EGFR Signaling
Source: PLoS One. 2010 Dec 14;5(12):e15341. doi: 10.1371/journal.pone.0015341 (PMC3001889; doi:10.1371/journal.pone.0015341)
Supplement: Materials and Methods S1 — Material and methods for apoptosis assays, single nsph gene profiling, neural colony forming cell assay and dissociated cell differentiation. (DOC) [file pone.0015341.s001.doc]

**Materials and Methods S1**

**Apoptosis assays**

Dissociated cells were plated onto PLL coated coverslips at 5x10^5^ cells/13 mm coverslip in GM containing chABC (20 mU/ml), chlorate (50 mM) or xyloside (150 μM) and incubated at 37ºC for 24 hours. Cells were fixed with 4% paraformaldehyde (PFA) and stained with DeadEnd Fluorometric TUNEL kit (Promega). Levels of active caspase 3/7 were evaluated using the Caspase-Glo 3/7 kit (Promega). Dissociated cells were plated into 96-well plates (NUNC) at bulk density with CSPG or CS-A to -E (50 μg/ml) for 24 hours, the Caspase-Glo reagent was added, and the relative active caspase 3/7 quantified.

**Single nsph gene profiling**

Individual control and CSPG (50 μg/ml) generated nsphs were placed directly into the reverse transcription reaction mix [gene specific primers (Taqman probes, Applied Biosystems), CellsDirect reaction mix and Taq polymerase (Invitrogen)] to pre-amplify genes of interest. The pre-amplified cDNAs were used for PCR reactions using BioMark chips (Fluidigm).

**Neural colony forming cell assay (NCFCA)**

Control and CSPG (50 μg/ml) nsphs generated from low-density cultures were harvested, dissociated into single cells and plated into the NCFCA collagen medium according to the manufacturer’s instruction (StemCell Technologies). Culture was continued for 3 weeks with weekly replenishment of medium and EGF. At the end of the culture period, colonies were counted and sized manually using the Olympus StereoZoom microscope. Nsphs from chABC (20 mU/ml), chlorate (50 mM) or xyloside (150 μM) treated bulk-density cultures were also harvested for this assay in the same way with untreated bulk-density cells as control.

**Dissociated cell differentiation**

ESC-derived nsphs were dissociated into single cells and seeded onto PLL and laminin coated glass coverslips at a density of 5x10^5^ cells/13 mm coverslip. Cells were cultured in differentiation medium for 7 days with half medium changes every 2 days.
