# Supplementary figures and images for: CSPG Is a Secreted Factor that Stimulates Neural Stem Cell Survival Possibly by Enhanced EGFR Signaling
Source: PLoS One. 2010 Dec 14;5(12):e15341. doi: 10.1371/journal.pone.0015341 (PMC3001889; doi:10.1371/journal.pone.0015341)

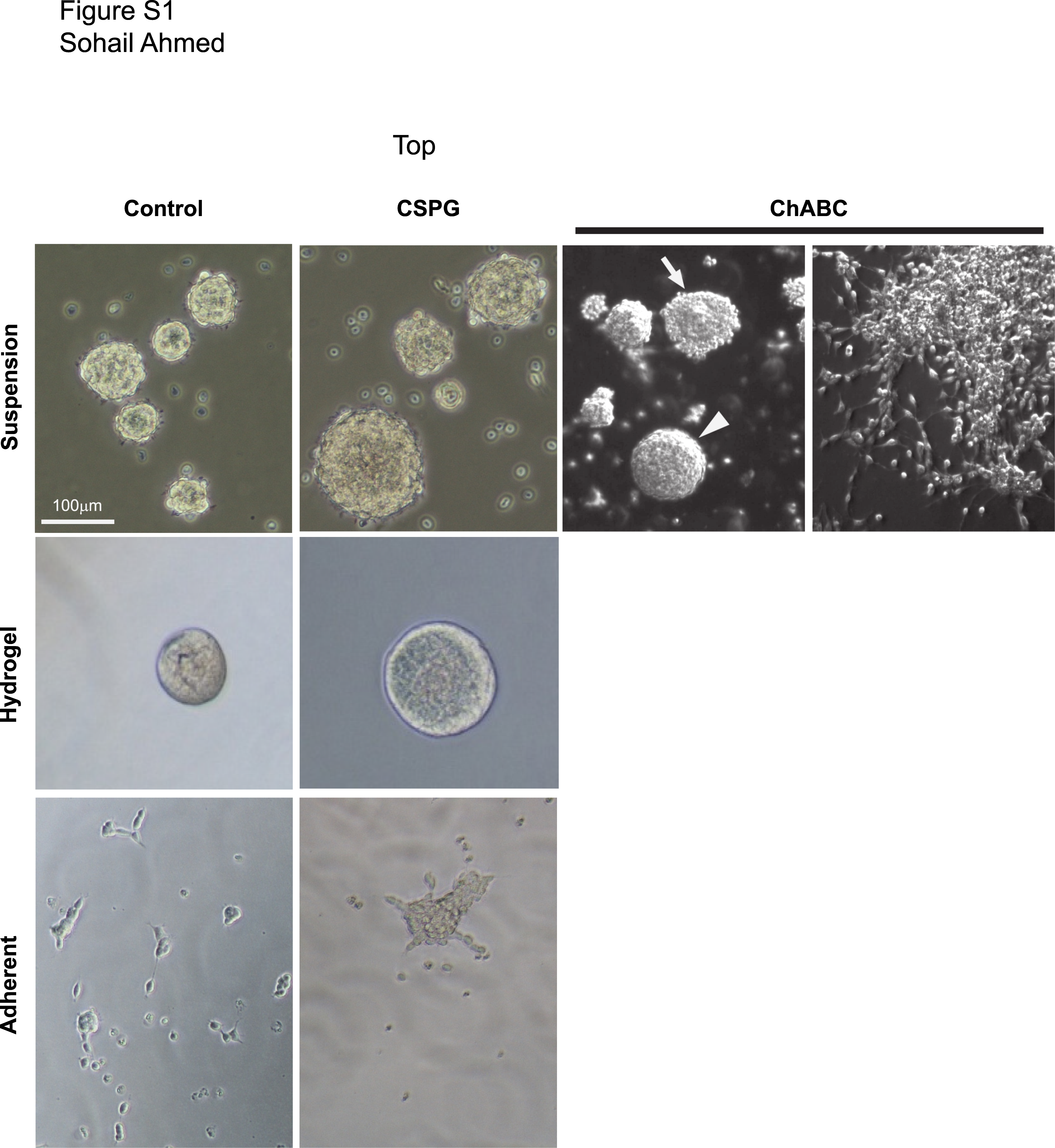

Supplement: Figure S1 — Images of cell culture systems. Dissociated cells were cultured in suspension, in hydrogel or on poly-L-lysine coated dishes to form adherent cultures. For control and CSPG (50 µg/ml) treated cultures, cells were plated at 2×10^3^ cells/ml. CSPG treatment stimulated proliferation in all culture conditions. For chABC (20 mU/ml) treated cultures, cells were plated at 2×10^4^ cells/ml. ChABC brokedown the 3D nsph into loss cell clusters (arrow, left panel) and induced cell attachment and proliferation as an adherent layer (right panel). Arrow head in the left panel marks a normal nsph. Scale bar = 100 µm at 10X objective. (TIF) [file pone.0015341.s002.tif]

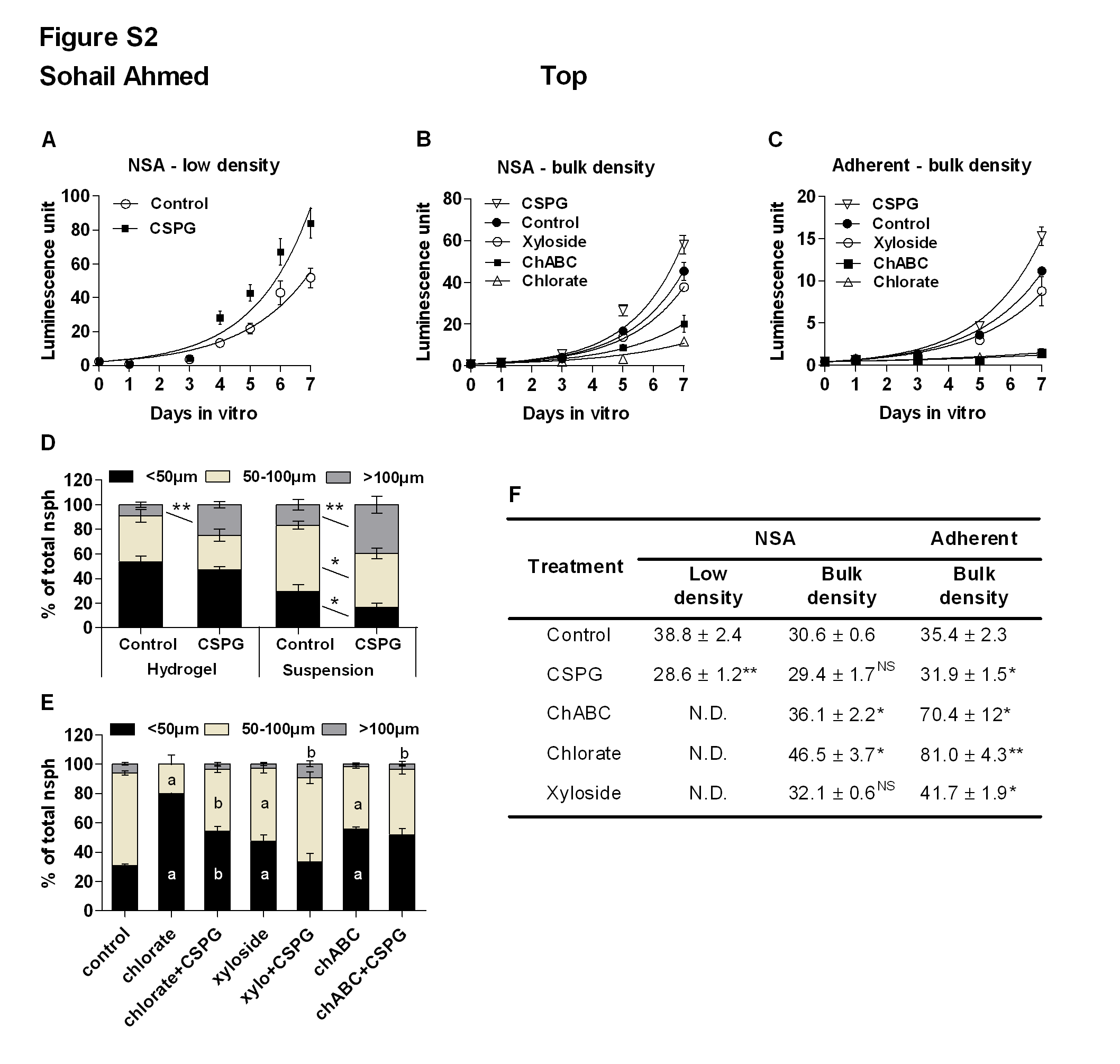

Supplement: Figure S2 — CSPG treatment increased NSC/NP proliferation. Cells were cultured in suspension at 2×10^3^ cells/ml (A) and 2×10^4^ cells/ml (B) or as adherent culture (C; 1×10^4^ cells/ml). The treatment conditions were CSPG (50 µg/ml), chABC (50 mU/ml), sodium chlorate (20 mM) or xyloside (150 µM). Total viable cells were determined on alternate days with the CellTitre Glo assay kit (Promega) measuring ATP level in viable cells by bioluminescence signals. Graphs show luminescence level against days in vitro. (D) and (E) The diameter of nsphs grown with and without CSPG (50 µg/ml), chABC (50 mU/ml), sodium chlorate (20 mM) or xyloside (150 µM) in suspension culture were measured and divided into three categories, <50 µm, 50–100 µm and >100 µm. Graph show percentage of nsph in each size category. For (D) nsph diameter was also measured in hydrogel. (F) Population doubling time (hours) for cells cultured under different conditions calculated from the above experiments using the GraphPad Prism software. Data are presented as mean ± SEM with n≥6 (from 2–6 experiments); *P≤0.05, **P≤0.01 compared to the control in each size category for (D) and in each density for (F); a = P≤0.01 compared to control and b = P≤0.01 compared to inhibitor only cultures in (E); N.D., not determined; NS, not significant. (TIF) [file pone.0015341.s003.tif]

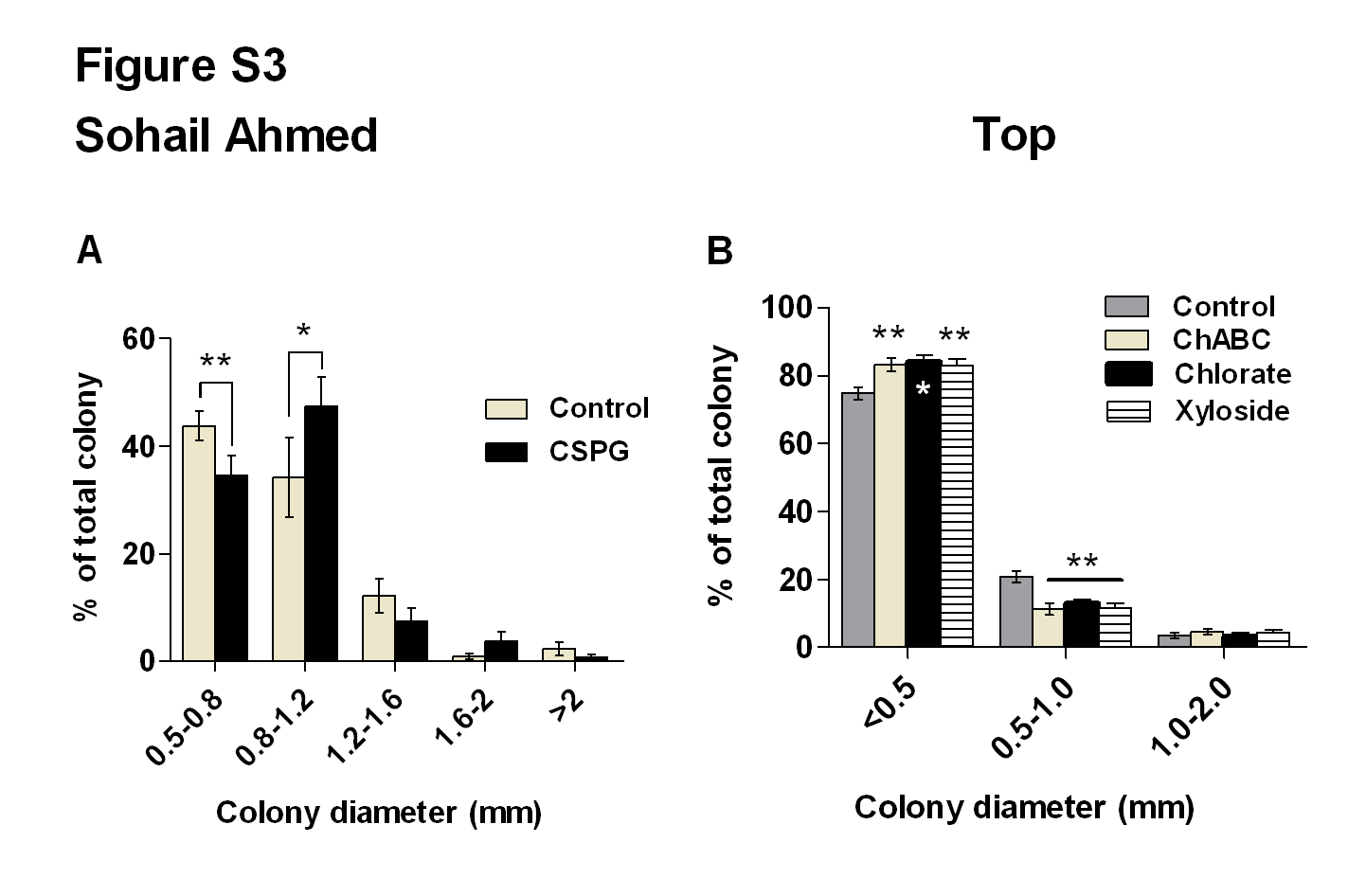

Supplement: Figure S3 — Neural colony forming assay. (A) Nsphs grown with or without CSPG (50 µg/ml) for seven days were dissociated and plated at 2.5×10^3^ cells/ml in NCFCA collagen medium. Colony number and size were recorded after three weeks. The bar chart shows the percentage of colonies above 0.5 mm in diameter. (B) Nsphs grown with or without chABC (50 mU/ml), sodium chlorate (20 mM) or xyloside (150 µM) for four days were analyzed as in (A). The bar chart shows the percentage of colonies in each size category. Data are presented as means ± SEM; n≥20 (from 5 experiments); * P≤0.05, ** P≤0.01 as compared to control, bar represent same significance level compared to control. (TIF) [file pone.0015341.s004.tif]

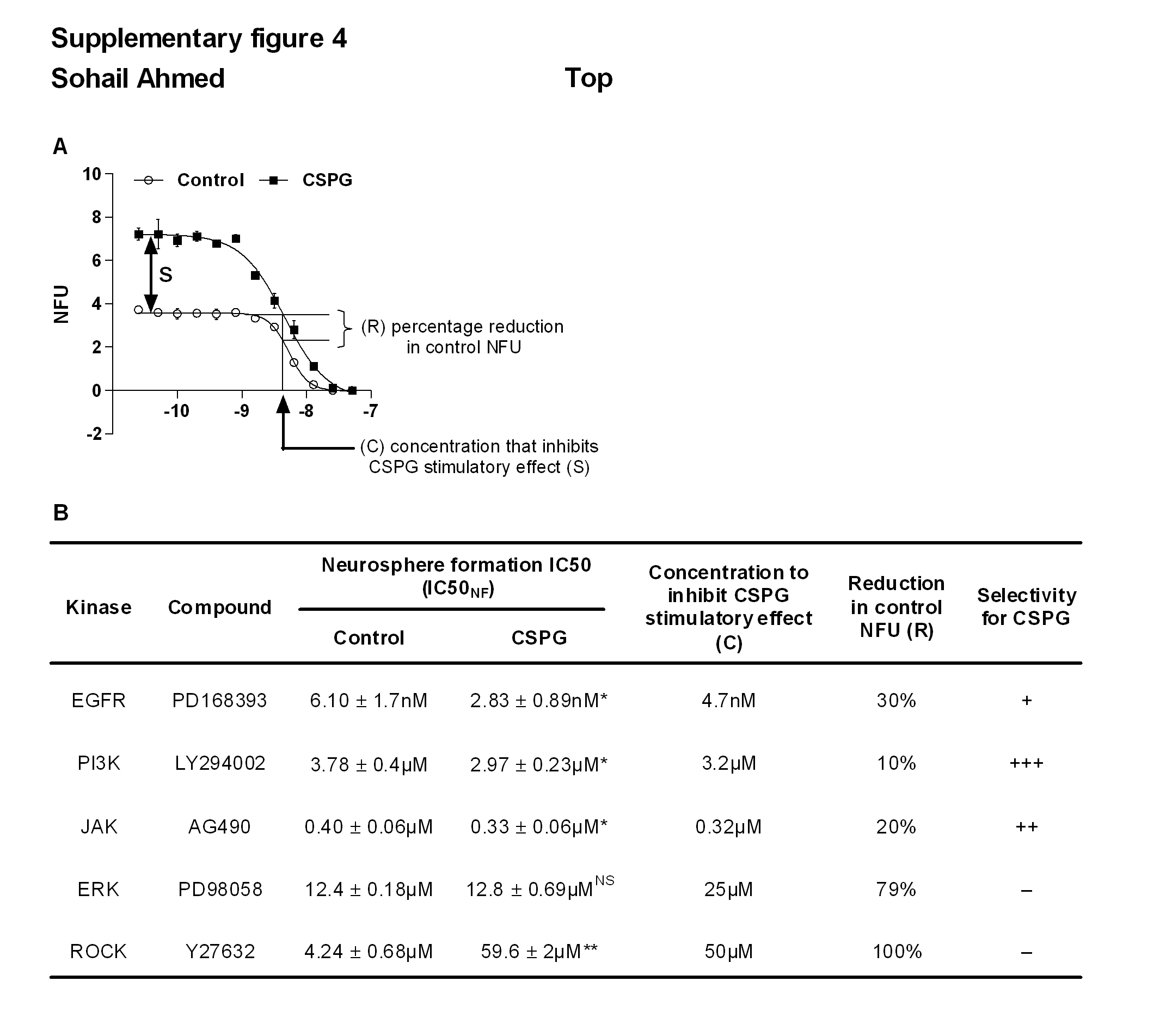

Supplement: Figure S4 — Interpretation of chemical inhibition data. (A) Diagram illustrating estimation of the inhibitor concentration that abolishes the stimulatory effect of CSPG (S) and its effect on control nsph formation rate. (B) Table summarizing the estimations from diagram (A) for each inhibitor as well as the IC50 values for nsph formation (IC50NF). Data are presented as means ± SEM with n = 9 (from 3 experiments); *P≤0.05, **P≤0.01 compared to control; NS, not significant. (TIF) [file pone.0015341.s005.tif]
